# Pediatric-related post-COVID condition (long COVID) research and its foundational influences: a bibliometric analysis (2020–2025)

**DOI:** 10.3389/fped.2026.1677983

**Published:** 2026-03-02

**Authors:** Natalya Chagay, Amin Tamadon, Svetlana Kim, Arystan Dossimov, Zhamilya Issanguzhina, Gulzhan Tulegenova, Gulmira Kuldeeva, Natalya Puxovikova, Irina Kim, Nadiar M. Mussin, Ramazon Safarzoda Sharoffidin

**Affiliations:** 1 Department of Children Diseases No. 2, West Kazakhstan Marat Ospanov Medical University, Aktobe, Kazakhstan; 2 Department of Natural Sciences, West Kazakhstan Marat Ospanov Medical University, Aktobe, Kazakhstan; 3 Department of Children Diseases No. 1, West Kazakhstan Marat Ospanov Medical University, Aktobe, Kazakhstan; 4 Department of Surgery No. 2, West Kazakhstan Marat Ospanov Medical University, Aktobe, Kazakhstan; 5 Department of Pharmaceutical Technology, Avicenna Tajik State Medical University, Dushanbe, Tajikistan

**Keywords:** bibliometric analysis, children, long COVID, pediatric complications, post-COVID syndrome

## Abstract

**Background:**

The COVID-19 pandemic significantly influenced healthcare systems worldwide. The long-term consequences of the infection in children, the phenomenon of post-COVID-19 syndrome, have been attracting increasing attention of the scientific community. The present study is a bibliometric analysis of publications addressing post-COVID (long COVID) complications in pediatric population over the period 2020–2025.

**Methods and materials:**

The analysis covers 1,292 records retrieved from Scopus and Web of Science (search date: June 2025). Records were retrieved using post-COVID condition/long COVID terminology combined with pediatric-related keywords; therefore, the corpus includes pediatric-focused studies as well as influential general PCC publications indexed with pediatric terms and frequently cited in pediatric research. The search strategy combined post-COVID condition/long COVID terminology with pediatric terms (child/infant/adolescent), applying filters for English language, publication years 2020–2025, and document type (articles and reviews). Data were merged and analyzed in R using bibliometrix/Biblioshiny to describe productivity, collaboration, citations, and thematic structure.

**Results:**

The retrieved corpus included 1,292 publications from 84 countries/regions. The United States led productivity with 270 publications (20.9%), followed by the United Kingdom (114; 8.8%) and China (90; 7.0%). The most frequent author keywords included “COVID-19” (*n* = 900) and “long COVID” (*n* = 818). Highly cited items predominantly consisted of general or mixed-age PCC frameworks, indicating that foundational long COVID literature substantially shapes citation patterns within pediatric-tagged publications. Thematic mapping showed symptom-focused clusters as dominant, while MIS-C and cognitive impairment were less prominent in author-keyword frequency and thematic clustering within the retrieved dataset.

**Conclusion:**

The findings describe the pediatric-term–indexed PCC research landscape and highlight substantial gaps in pediatric-specific evidence, definitions, and longitudinal data.

## 1 Introduction

The coronavirus infection, whose first cases were identified in December 2019, rapidly disseminated across the globe and was declared a pandemic within three months (1–6). Children were initially considered to be less susceptible to Sars-CoV2. However, evidence from contact-tracing and population studies suggests that susceptibility to SARS-CoV-2 infection differs by age, with children and adolescents generally showing lower susceptibility than adults in meta-analytic estimates, although findings vary by setting and pandemic phase (7).

Since the COVID-19 pandemic, many cases of prolonged COVID-19 have been identified (8), which has attracted the attention of many researchers from around the world. Estimates of persistent symptoms after SARS-CoV-2 infection have been reported at large scale, largely reflecting adult and mixed-age populations; however, pediatric-specific prevalence and clinical phenotypes vary across settings and definitions. This underscores the need to map pediatric research output and thematic priorities separately (9).

The World Health Organization has formulated a definition: “post-COVID syndrome” or “long COVID” is a condition that can manifest in people of any age, regardless of the severity of the SARS-CoV-2 infection. This condition develops no earlier than three months after primary infection and persists for at least two months with no other alternative diagnosis (10).

WHO estimates that approximately 10%–20% of infected may develop post-COVID-19 condition (11). According to U.S. epidemiological data, in 2022 6.9% of adults and 1.3% of children (approximately 17 million and 1 million people, respectively) reported experiencing symptoms of prolonged COVID (12).

Although several bibliometric studies have examined long COVID research broadly, pediatric-focused mapping remains limited. The present study contributes novelty by: (i) focusing on children and adolescents, (ii) covering the 2020–2025 period capturing early and evolving PCC literature, (iii) integrating both Web of Science and Scopus records to expand coverage, and (iv) providing combined analyses of productivity, collaboration networks, citations, and thematic structure to identify dominant and under-represented themes in pediatric PCC research. Similar bibliometric approaches have been applied in pediatric COVID-19 subfields, supporting the utility of bibliometrics to identify research gaps and collaboration patterns (13).

In this manuscript, we use the term post-COVID-19 condition (PCC) [also referred to as long COVID or post-acute sequelae of SARS-CoV-2 infection (PASC)]. While these labels are sometimes used interchangeably across studies, they broadly describe persistent or new symptoms following acute SARS-CoV-2 infection. This bibliometric analysis aims to assess the volume, geographic distribution, and temporal evolution of global scientific literature on post-COVID-19 condition (long COVID) in children and adolescents from 2020 to 2025. It maps research productivity, collaboration networks, citation patterns, and thematic structure to identify dominant and under-represented topics in pediatric PCC research.

## 2 Materials and methods

Data were obtained from the Scopus and Web of Science databases using advanced search options. We focused on Scopus and Web of Science because they provide standardized citation metadata and broad cross-disciplinary coverage suitable for citation-network and source analyses; however, we acknowledge that PubMed/MEDLINE contains additional biomedical journals and its exclusion may lead to coverage bias. We included original and review articles published in English in 2020–2025. Articles and reviews were analyzed together because both contribute to the scientific discourse and citation structure. However, we recognize that reviews and consensus statements may amplify visibility of specific primary studies through repeated citation. To address this, we report document type composition and interpret productivity/citation findings with caution.

**Figure 1:**
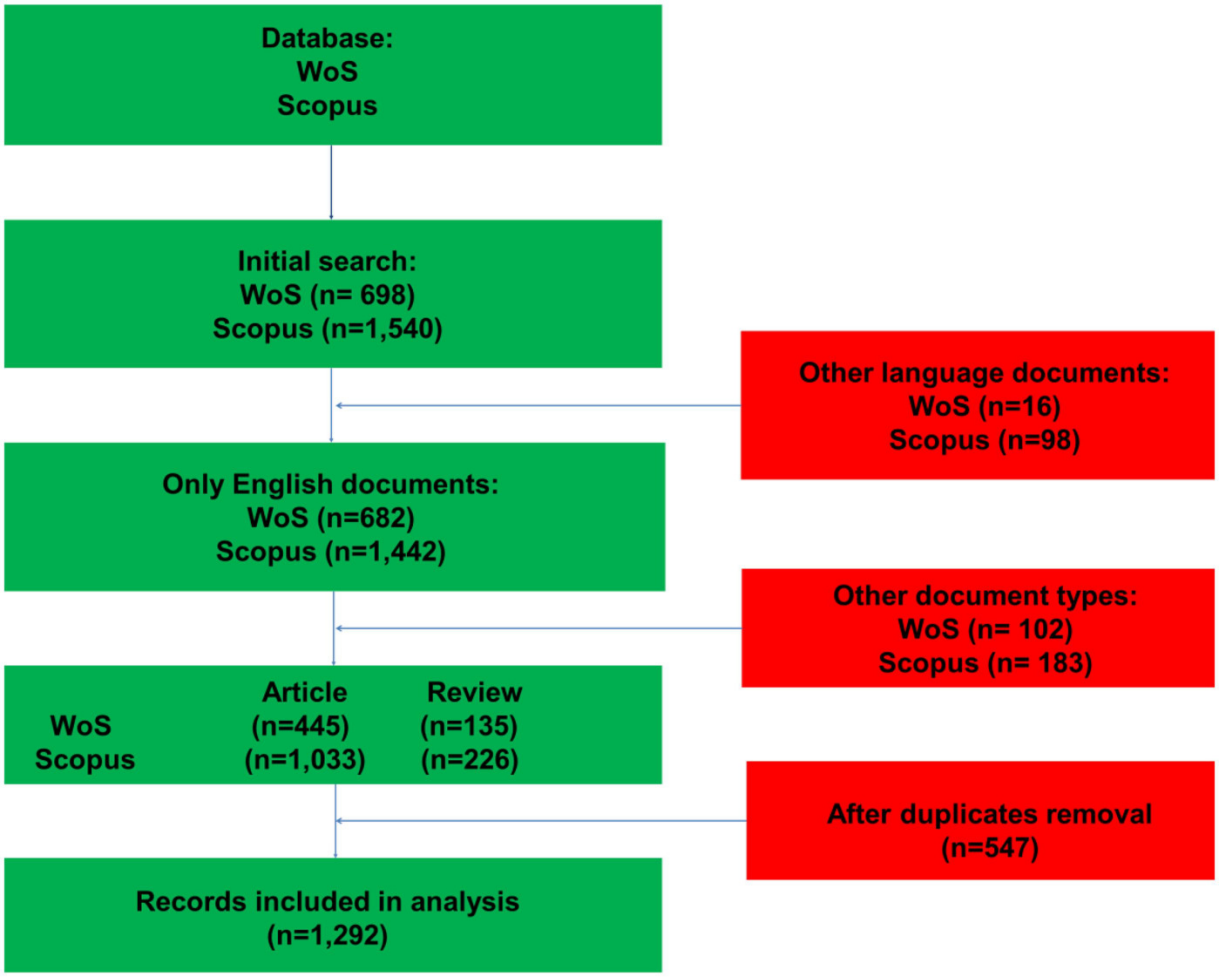
Flowchart for selecting records in the bibliometric analysis of post-COVID-19 complications in children.

All relevant metadata were obtained in BibTeX and text format, respectively. Data were merged in RStudio as a excel file (Table 1). A combination of Boolean and Wildcard search operators was utilized to systematically identify relevant keywords for the purposes of the study (Tables 2, 3). The search was conducted in June 2025, and the complete search strategy is detailed in Figure 1. After obtaining the results, 1,292 records for bibliometric analysis were imported. Data management and bibliometric analysis were conducted using the bibliometric package (version 4.1.3) and Biblioshiny web applications in RStudio (RStudio 2023.09.1+494, PBC, Boston, MA).

**Table 1:** Codes were used to merge Scopus and Web of Science exported data in RStudio.

RStudio code
library(bibliometrix) library(openxlsx) ## importing web of science dataset web_data<-convert2df(“abs.txt”) ## importing scopus dataset scopus_data<-convert2df(“abs.bib”,dbsource="scopus”,format="bibtex”) ##combined both datasets combined<-mergeDbSources(web_data,scopus_data,remove.duplicated=T) ##exporting file write.xlsx(combined,"combinedabs.xlsx”)

**Table 2:** Queries for selecting records in the bibliometric analysis of post-COVID-19 complications in children from Scopus database.

Query
(TITLE-ABS-KEY (“long COVID” OR “post-COVID condition” OR “post-COVID conditions” OR “long-haul COVID” OR “post-acute COVID-19” OR “long-term effects of COVID” OR “chronic COVID” OR “persistent COVID-19 symptoms” OR “post-COVID-19 manifestations” OR “post COVID-19 syndrome” OR “ongoing symptomatic COVID-19” OR “post-acute sequelae of SARS-CoV-2 infection” OR PASC) AND TITLE-ABS-KEY (pediatric* OR paediatric* OR child* OR infant* OR adolescen* OR teen* OR youth*) AND (LIMIT-TO (LANGUAGE, “English”)) AND (LIMIT-TO (DOCTYPE, “ar”) OR LIMIT-TO (DOCTYPE, “re”)) AND (PUBYEAR > 2019 AND PUBYEAR < 2026)

**Table 3:** Queries for selecting records in the bibliometric analysis of post-COVID-19 complications in children from Web of Science database.

No	Queries
#1 (PCC terms in Topic fields)	TS=(“long COVID” OR “post-COVID condition*” OR “long-haul COVID” OR “post-acute COVID-19” OR “long-term effects of COVID” OR “persistent COVID-19 symptom*” OR “post-acute sequelae of SARS-CoV-2 infection” OR PASC)
#2 (Pediatric terms in Topic fields)	TS=(pediatric* OR paediatric* OR child* OR infant* OR adolescen* OR teen* OR youth*)
#3	#1 AND #2 Refined by: LANGUAGE: English; DOCUMENT TYPES: Article OR Review; PUBLICATION YEARS: 2020–2025

Duplicates were identified and removed by bibliometrix merge rules, primarily matching DOI when available and otherwise using title/author/year metadata; records with identical identifiers across databases were retained only once.

We reported the study following relevant guidance for bibliometric reporting and transparent workflow description (including database query reporting, merging, and analytical steps), and we present the record selection process using a flow diagram consistent with scoping-style reporting.

We included peer-reviewed articles and reviews published in English between 2020 and 2025 and indexed in Scopus or Web of Science. We excluded non-article/review document types to improve comparability of citation and keyword structures. Records in languages other than English were excluded. Because this is a bibliometric mapping study, “pediatric” was operationalized using pediatric-related search terms (child/infant/adolescent and pediatric synonyms), acknowledging that some mixed-age or general PCC papers may still be captured if indexed with pediatric terms. Because pediatric focus was operationalized through pediatric-related search terms rather than age-restricted inclusion, the retrieved corpus may include mixed-age or general PCC publications indexed with pediatric descriptors.

Prior to co-word analysis, author keywords were harmonized by converting to lowercase and merging close synonyms, while retaining clinically meaningful symptom terms. We applied a minimum-frequency threshold (reported in the Results/figure settings) and generated the thematic map using co-occurrence-based centrality and density measures in Biblioshiny. We acknowledge that generic indexing terms may remain and can influence clustering if not fully excluded.

## 3 Results

The flow chart (Figure 1) illustrates the data process. The analysis covered a 6-year period for articles and reviews, focusing on publication and citation metrics and citation trends (14). Prolific institutions were identified based on the number of articles related to the post-COVID-19 complications in children.

We analysed bibliometric indicators, including leading countries, leading journals, leading articles, and leading authors. A country collaboration map was generated. Keyword frequency, co-occurrence, and thematic mapping were performed to identify research hotspots and thematic structure. Through high-frequency keywords and keywords co-occurrence map hotspots were identified.

A TreeMap visualization depicted the 10 most frequently used keywords in articles published on this topic. Lastly, a Thematic Map categorized keywords into four domains: motor themes, basic themes, niche themes and emerging or declining themes, based on their centrality and density.

### 3.1 Leading countries

**Figure 2:**
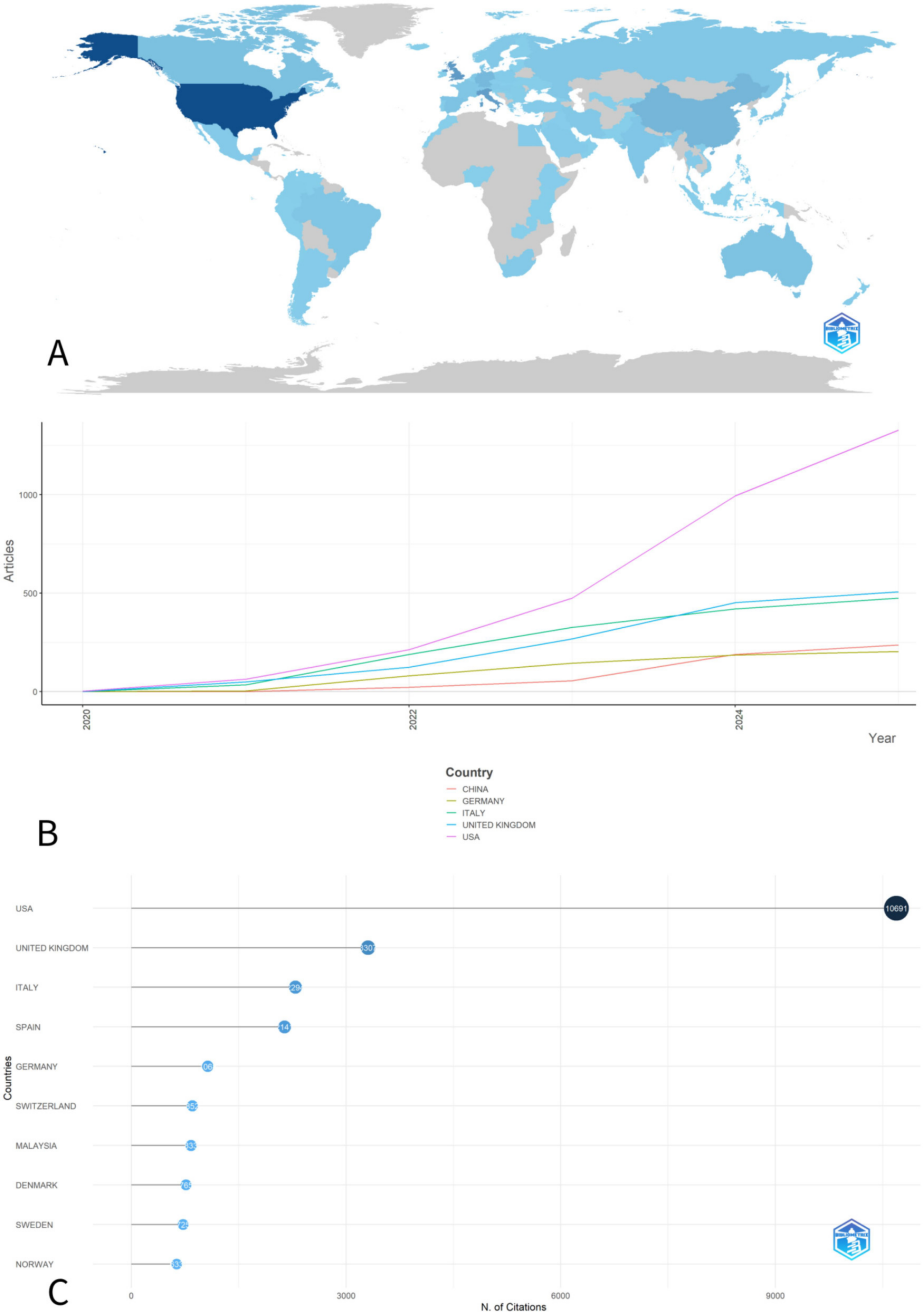
**(A)** Countries’ scientific production on children's complications in long COVID; **(B)** Top 5 countries of scientific production over 2020–2025 period; **(C)** 10 most cited countries within the PCC literature retrieved using pediatric-related search terms.

According to the country of origin of the corresponding authors, representatives of 84 countries and regions contributed to the publications included in the study. Depending on the number of publications, the top 10 productive countries were identified (Figure 2A). The United States was the leader with 270 publications (20.9%), followed by Italy (74, 5.7%), the United Kingdom (114, 8.8%), China (90, 7.0%) and Germany (64, 5.0%). According to Figure 2B, which depicts publication dynamics over 6-year period, all the mentioned above countries started publishing on topic in 2020. However, since 2021 the US has consistently maintained the leading position of publication volume. In terms of citations, the US also ranked first with a total of 10,691 citations and an average of 39.6 citations per article. It was followed by the UK (3,307), Italy (2,294), Spain (2,141) and Germany (1,068). It is worth noting that the United States and the United Kingdom together accounted for 30.3% of all publications and 48.26% of total citations, underscoring their central role in advancing research in this field. Country attribution was based on the corresponding author's country, which may underrepresent multinational contributions in multicenter studies where collaborators are located in other countries; this should be considered when interpreting geographic distributions.

### 3.2 Leading journals

**Figure 3:**
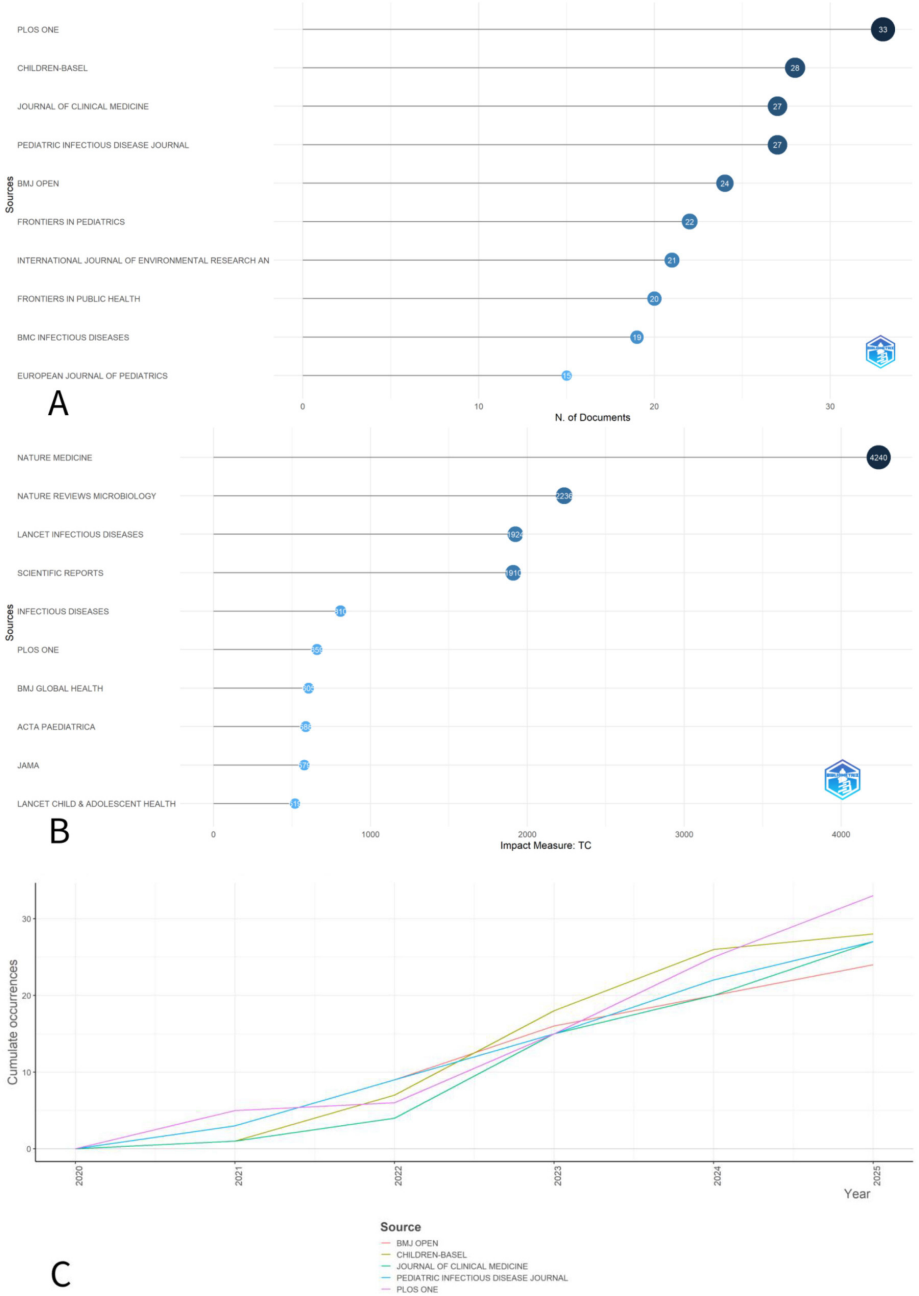
**(A)** Top 10 most productive journals on pediatric complications in post-COVID; **(B)** Top 10 journals with highest number of citations; **(C)** Sources production dynamics over 2020–2025.

The 1,292 publications entered this study were published in 615 journals. According to the number of publications, we listed the top 10 most productive journals (Figure 3). 33 articles were featured in the journal “PLOS ONE” followed by 28 records in the journal “CHILDREN-BASEL”. From the perspective of quoted quantity, “Nature Medicine” is considered number one, as it has 4,240 total citations. “Nature Reviews Microbiology” was the next, with 2,236 total citations. “Lancet Infectious Diseases” and “Scientific Reports” contributed 1924 and 1910 citations respectively. In terms of overtime production, “PLOS ONE”, “Pediatric Infectious Diseases” and “Journal of Clinical Medicine” were first to publish relevant scientific articles in 2020. In 2021 “CHILDREN-BASEL” joined the team and in 2022 – “BMJ Open”.

The most highly cited sources are not pediatric-only journals, indicating that high-impact general long COVID frameworks and consensus papers are heavily cited within pediatric-oriented publications. Thus, citation dominance likely reflects foundational long COVID references rather than exclusively pediatric clinical focus.

### 3.3 Leading authors

**Figure 4:**
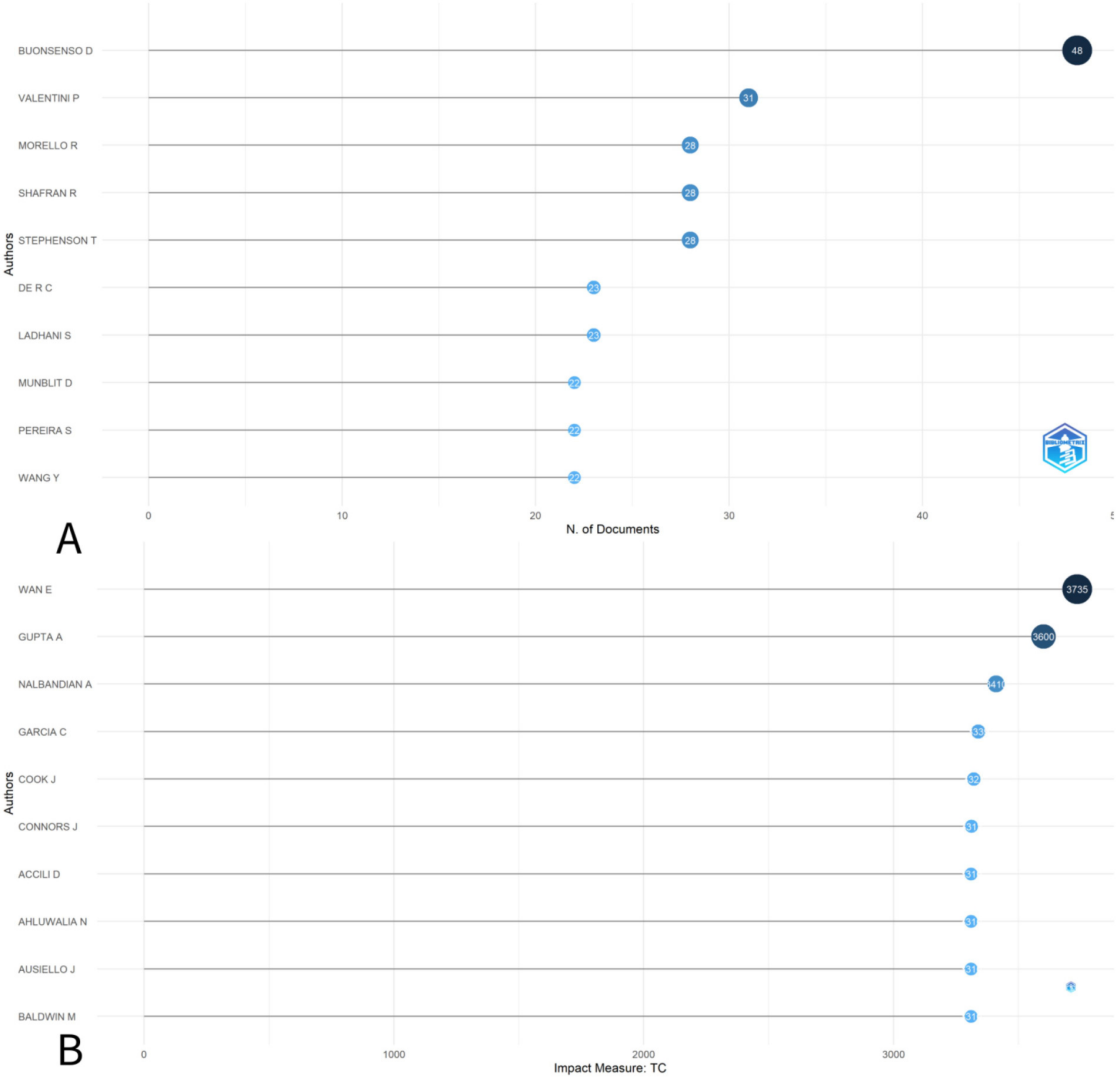
**(A)** Top 10 most prolific authors of articles in the field of pediatric complications in post-COVID; **(B)** Top 10 most cited authors on post-COVID complications in children.

A total of 10,593 authors contributed to the 1,292 publications included in this analysis. Among the most productive authors, Buonsenso D had the highest number of publications (*n* = 48), followed by Valentini P (*n* = 31), Morello R, Shafran R, and Stephenson T (*n* = 28 each). Highly cited authors included Wan E, Gupta A, and Nalbandian A, whose prominence reflects their contributions to influential general or mixed-age PCC frameworks (Figure 4). Because the dataset was constructed using pediatric-related search terms rather than age-restricted inclusion, author productivity and citation impact should be interpreted as reflecting the broader PCC research landscape indexed with pediatric descriptors, rather than pediatric-only research leadership.

### 3.4 Leading articles

Table 4 lists the top 10 articles based on citation rate. Most highly cited papers represent general or mixed-age PCC frameworks rather than pediatric-exclusive studies, reflecting the foundational influence of broader long COVID literature on pediatric-tagged research. The most total cited publication was “Post-acute COVID-19 syndrome” written by Nalbandian A et al., which reviews the long-term effects of COVID-19, detailing persistent symptoms across multiple organ systems and proposing diagnostic and management strategies. The second publication - by Davis H et al. – is two years younger than the previous one, but possesses more citations per year. The total citations of all 1,292 publications accounted to 30,052 and the average citation number was 23.26 per article. Among them, the top 10 publications had 12,282 citations and contributed 40.86% of the total citations.

**Table 4:** Top 10 most cited articles within the post-COVID condition corpus retrieved using pediatric-related search terms (2020–2025).

Rank	Title	Author	PY	Total Citations	TC per Year
1	Post-acute COVID-19 syndrome	Nalbandian A	2021	3,310	662.00
2	Long COVID: major findings, mechanisms and recommendations	Davis H	2023	2,236	745.33
3	A clinical case definition of post-COVID-19 condition by a Delphi consensus	Soriano J	2022	1,914	478.50
4	More than 50 long-term effects of COVID-19: a systematic review and meta-analysis	Lopez-Leon S	2021	1,556	311.20
5	Long COVID or post-COVID-19 syndrome: putative pathophysiology, risk factors, and treatments	Yong S	2021	810	162.00
6	Characterising long COVID: a living systematic review	Michelen M	2021	575	115.00
7	Estimated global proportions of individuals with persistent fatigue, cognitive, and respiratory symptom clusters following symptomatic COVID -19 in 2020 and 2021	Vos T	2022	573	143.25
8	Long COVID in a prospective cohort of home-isolated patients	Blomberg B	2021	473	94.60
9	Immune determinants of COVID-19 disease presentation and severity	Brodin P	2021	457	91.40
10	Preliminary evidence on long COVID in children	Buonsenso D	2021	378	75.60

Several top-cited papers are broad PCC/long COVID syntheses rather than pediatric-only studies. This is expected in bibliometric datasets built on keyword indexing, because general high-impact PCC frameworks are frequently cited by pediatric publications and may be indexed with pediatric-related terms.

### 3.5 Tree map and thematic map

**Figure 5:**
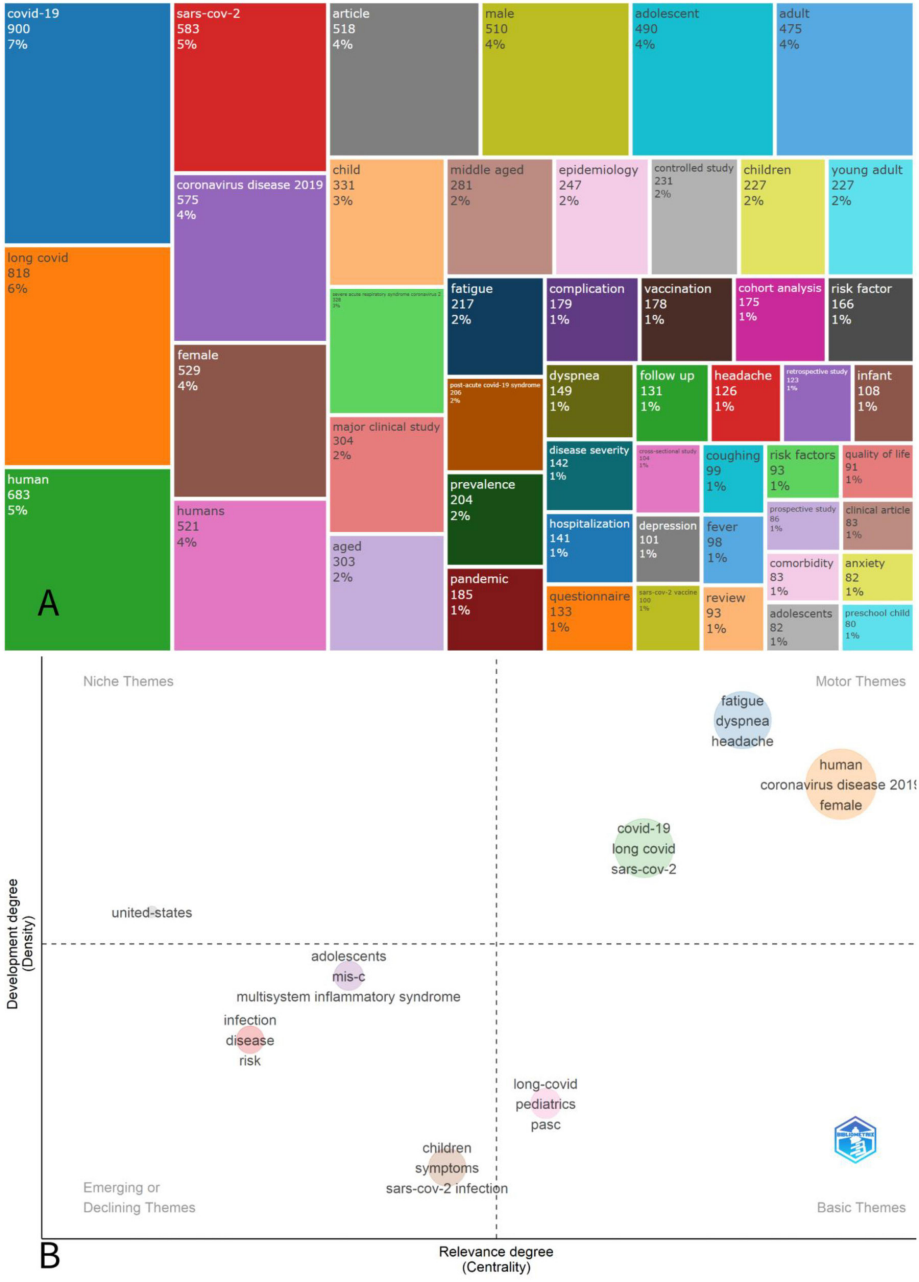
**(A)** A tree map representing 20 most relevant keywords found in the articles on pediatric complications in post-COVID. **(B)** A thematic map presenting clusters of keywords based on their centrality (relevance to the field) and density (internal development).

Figure 5A is a TreeMap visualization that highlights 20 the most frequently used keywords in scientific publications on the pediatric post-COVID complications. “Covid-19” was the most common keyword with its frequency 900 times, followed by “long covid” with 818 times. The next were “human” (683) and “Sars-Cov-2” (583). The prominence of generic terms such as “human”, “female”, and “adult” likely reflects indexing conventions and residual keyword noise, and therefore should be interpreted as methodological artifacts rather than pediatric-specific thematic priorities. The presence of non-pediatric demographic terms such as ‘adult’ and ‘female’ reflects database indexing and citation of general PCC literature within pediatric-tagged records rather than true pediatric thematic dominance.

The thematic map (Figure 5B) was based on co-word analysis of author keywords. It presents a two-dimensional graph that measures centrality (*X*-axis, signifying the topic's significance) and density (*Y*-axis, indicating theme development). The map is divided into four quadrants, each representing a different thematic typology: Quartile 1 (Q1) – motor themes – are well-developed and important themes for structuring the research field. Q1 includes two major thematic areas: one cluster consisting of “human”, “female” and “coronavirus disease 2019” suggesting an emphasis on general population-level studies; another cluster focused on specific long COVID symptoms, including “fatigue”, “dyspnea” and “headache”, reflecting high interest in the clinical manifestations of post-acute sequelae. In contrast, Q2 – basic themes - represents fundamental and transversal topics that are important to the field but are less developed. The cluster here includes “long-COVID”, “pediatrics”, “PASC”, “children” and **“**symptoms” indicating under-explored areas in pediatric long COVID research. Q3 – emerging or declining themes - signifies emerging or waning themes. This quadrant includes “MIS-C,” “multisystem inflammatory syndrome”, “infection”, “disease”, and “risk” suggesting topics that may represent earlier pandemic concerns or niche clinical subtopics requiring renewed research attention. And the last quadrant – niche themes – possesses only one word “united states” suggesting region-specific focus without broader thematic influence. The size of each circle corresponds to the frequency of the occurrence of the respective term. Additionally, the hierarchical arrangement of density and centrality ranks can be showed as follows: Q1 > Q4 > Q3 > Q2 for density. Meanwhile, for centrality, the order is Q1 > Q2 > Q3 > Q4, again indicating a descending order of ranks from high to low.

### 3.6 Three-fields plot

**Figure 6:**
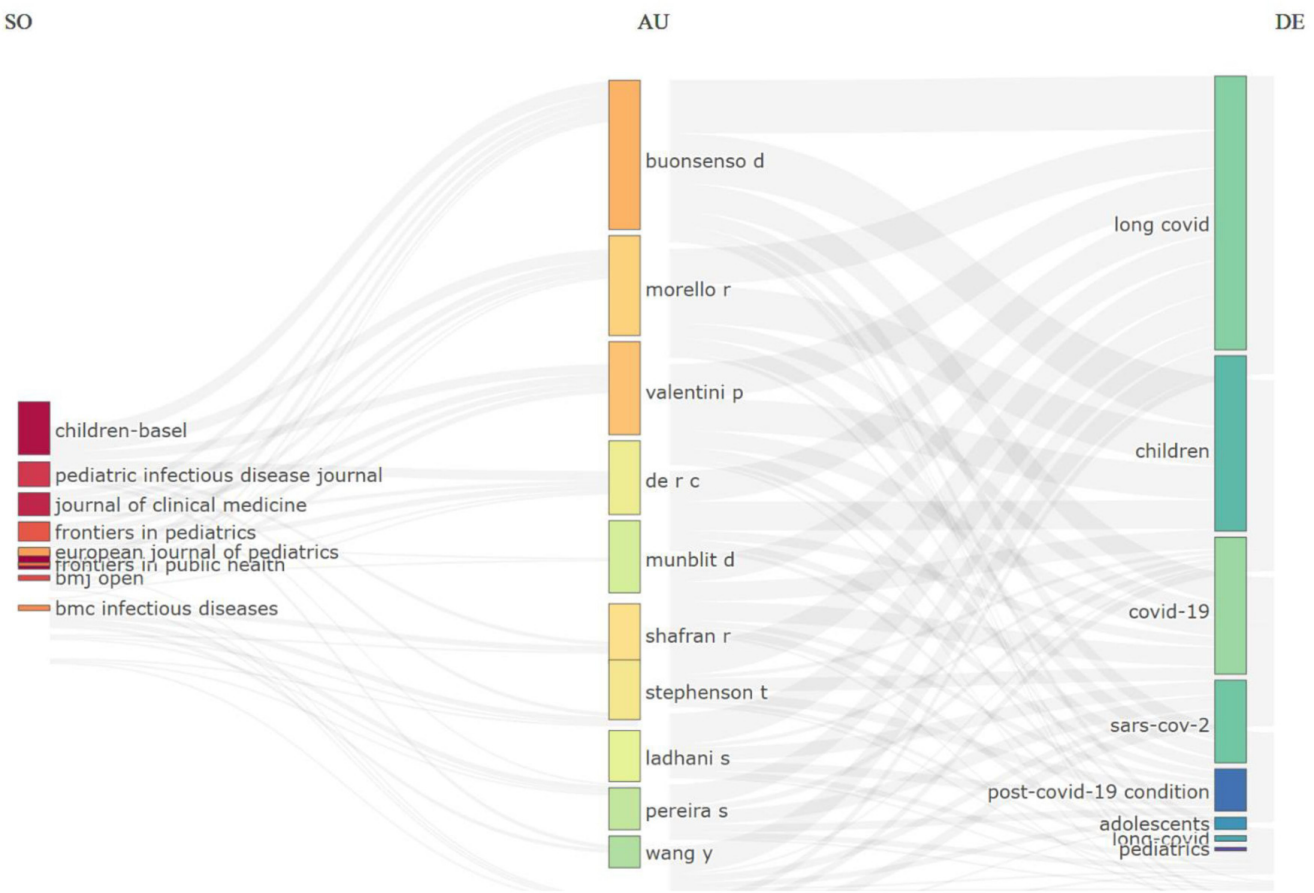
A three-fields plot illustrating the interconnections between the top ten journals, authors, and keywords that have made contributions to articles on pediatric complications in post-COVID. This plot visualizes the incoming and outgoing flows of influence among these key elements in the research field.

To visualize the contributions of the top 10 journals, authors, and keywords related to the pediatric complications in post-COVID, a graphical representation was developed highlighting the interconnections among these elements (Figure 6).

## 4 Discussion

An important interpretive finding of this bibliometric analysis is the strong influence of general and adult-focused PCC frameworks on pediatric-tagged literature. Highly cited definitions, reviews, and consensus papers that explicitly include, exclude, or call for pediatric-specific research are frequently cited within child-related publications. This dependency does not imply equivalence between adult and pediatric evidence; rather, it highlights the current lack of pediatric-specific definitions, standardized outcomes, and long-term cohorts, reinforcing the need for dedicated pediatric PCC research.

### 4.1 Contribution of countries, journals, and authors

The bibliometric analysis revealed that research on post-COVID complications in children is a globally distributed field, with contributions from 84 countries and regions. According to the analysis the US and European countries are dominant contributors in terms of both publication volume and citation frequency. This aligns with previous findings suggesting that countries with historically well-established scientific infrastructures, sustained investment and high concentrations of talented scholars are more likely to produce influential scientific output (14–16). Furthermore, the US and the EU lead in international collaborative authorship. As Leydesdorff et al. claims internal collaboration in the EU contributes approximately 1% to the global total citation rate and enhances region's scientific impact (17). According to Man et al., English level proficiency largely determines publication acceptance by leading medical journals (18).

From the perspective of the number of articles, publications on the complications of long-term COVID in children were disseminated across 615 different scientific journals, with no concentration in a specific publication outlet. The widespread distribution indicates interdisciplinary interest in the topic, spanning pediatrics, infectious diseases, immunology and public health. “Nature Medicine” and “Nature Reviews Microbiology” achieved the best results in terms of citation rates, despite not being among the top 5 journals by number of publications. Tahamtan et al. states that journal impact factor, number of authors, prominence and international collaboration are the strongest predictors of high citation performance (19). Conversely, “PLOS ONE” and “CHILDREN-BASEL” were the most productive in terms of article volume, reflecting both the openness of these platforms and the diversity of study types. Lewis and Davis suggest that Open Access publishing significantly increases citations rate (20, 21), while Basson et al. conclude that access status alone has limited or no effect when controlling for other variables (22).

Authorship patterns indicated that a small number of authors contributed multiple publications within the retrieved corpus. However, because pediatric focus was operationalized through pediatric-related search terms rather than age-restricted inclusion, highly cited and highly productive authors may reflect the broader PCC research ecosystem indexed with pediatric descriptors. Therefore, author prominence should not be interpreted as pediatric-only research leadership, but rather as an indicator of the foundational influence of general and mixed-age PCC literature on pediatric-tagged publications.

Interpretation of author prominence and collaboration patterns in this study requires caution. Because pediatric focus was operationalized through search terms rather than age-restricted inclusion, highly cited authors and collaborative structures primarily reflect the broader post-COVID condition research ecosystem. Foundational adult or mixed-age PCC frameworks are frequently cited within pediatric-tagged publications and therefore exert a strong influence on authorship visibility. This should not be interpreted as evidence of pediatric research dominance by adult-focused groups, but rather as an indicator of the current reliance of pediatric PCC research on general conceptual frameworks and the relative immaturity of pediatric-specific collaborative networks.

### 4.2 Analysis of research hotspots

The TreeMap and Thematic Map analyses reveal key research hotspots in pediatric post-COVID complications. The prominence of keywords like “COVID-19,” “long COVID,” and “SARS-CoV-2” in the TreeMap reflects sustained interest in pathophysiology and long-term sequelae of SARS-CoV-2 infection. Complementarily, the thematic map confirmed that the most intensively investigated symptoms, such as “fatigue”, “dyspnea” and “headache”, are strongly associated with pediatric long COVID. These terms fell into Q1 (motor themes), confirming their importance and active study (8, 23, 24). From a research-trend perspective, symptom clusters such as fatigue, dyspnea, and headache emerge as dominant themes, suggesting these areas have attracted sustained investigation and may represent priority targets for future pediatric PCC studies and standardized outcome reporting. In contrast, key words as “pediatrics”, “PASC” and “symptoms” were identified as core but underexplored, suggesting the need for more in-depth research and standardized definitions in the pediatric population.

The Three-Fields Plot reveals the structural and collaborative dynamics of pediatric long COVID research activity. The graph demonstrates prolific authors’ influential role, such as Buonsenso D, who contributed to high-impact journals and focused on keywords like “long COVID” and “children”. These findings align with Jin et al., who emphasize the importance of keyword co-occurrence and author collaboration for identifying research trends and underexplored areas in long COVID studies (15).

### 4.3 Research opportunities

Despite a growing number of literatures due to the relatively novel clinical condition, some critical issues remain unaddressed. For example, the disproportionate citation rate of certain countries and journals raises questions of equity regarding global representation on pediatric post-COVID. Furthermore, the isolation of author groups points to the potential for increased international collaboration, particularly in low- and middle-income countries. Conducting multinational studies could greatly increase the diversity and applicability of findings.

There is also a lack of studies focusing on multisystem inflammatory syndrome (MIS-C) and broader complications of COVID. Addressing this disconnect through comparative studies or integrated meta-analyses could lead to a more comprehensive understanding of pediatric post-COVID sequelae. Importantly, MIS-C and post-COVID condition/long COVID represent overlapping but conceptually distinct post-infectious entities: MIS-C is typically an acute/subacute hyperinflammatory syndrome temporally linked to SARS-CoV-2 exposure, whereas PCC emphasizes persistent or relapsing symptoms extending beyond the acute phase. Therefore, a lower prominence of MIS-C within PCC-themed keyword clusters should not be interpreted as lack of MIS-C research overall, but rather as a thematic separation between MIS-C and PCC research streams.

Equally important is the interdisciplinary nature of pediatric post-COVID research that will open opportunities for collaboration with diverse fields such as psychology and neuroscience to study the cognitive and psychosocial effects of long-term COVID in children. These kinds of interdisciplinary initiatives could be directed at understudied areas, particularly those related to school performance or mental health. We agree that explanatory modeling could provide deeper insight into drivers of productivity and research disparities. Such analyses require harmonized epidemiological denominators across countries and were beyond the descriptive scope of the present bibliometric mapping; we propose this as an important next step.

### 4.4 Limitations

Despite providing a comprehensive bibliometric mapping of post-COVID-19 condition (PCC)/long COVID research in children, several limitations should be acknowledged. First, combining Scopus and Web of Science may introduce coverage bias due to differences in indexing scope and metadata structure, even after duplicate removal. In addition, PubMed/MEDLINE was not included; therefore, some pediatric clinical journals and region-specific biomedical publications may be underrepresented, potentially influencing country, source, and keyword distributions. Second, the pediatric focus was operationalized using pediatric-related search terms rather than an explicit age filter; consequently, some mixed-age or general PCC records may have been captured if indexed with pediatric terms. Third, country attribution was based on the corresponding author's affiliation, which may underrepresent multinational contributions in multicenter studies. Fourth, although we performed keyword harmonization, residual generic indexing terms may persist and influence co-word clustering; thematic-map outputs may also vary depending on the chosen frequency thresholds and cleaning parameters. Accordingly, collaboration patterns were not interpreted as pediatric-specific research networks, because co-authorship structures in the retrieved corpus are influenced by broadly cited general PCC literature. Fifth, the corpus included both primary studies and secondary literature (reviews/meta-analyses/consensus papers); therefore, citation-based indicators and author prominence may be influenced by secondary works that repeatedly cite the same primary evidence base, potentially inflating the perceived influence of certain topics or authors. Document-type–stratified analyses (articles-only vs. reviews-only) could be considered as a sensitivity approach in future work. Sixth, we did not perform inferential or burden-normalized analyses; therefore, observed geographic patterns should not be interpreted as proportional to pediatric disease burden or research investment. Finally, we restricted inclusion to English-language publications to improve consistency of indexing and keyword comparability across databases and due to practical constraints in translation and normalization of non-English records. This decision may underrepresent scientific output from non-English-speaking regions and may affect geographic and thematic patterns.

## 5 Conclusion

The conducted bibliometric analysis represents the significant and constantly growing interest of post-COVID complications in children. The main contribution to the topic is made by the USA and European countries, demonstrating both quantitative and qualitative leadership. The analysis identified leading contributing countries and influential sources within the retrieved pediatric-term–indexed PCC corpus, while emphasizing that citation visibility is strongly shaped by general PCC frameworks. Citation patterns emphasize symptom-focused PCC research driven largely by general frameworks, while pediatric-specific entities such as MIS-C, neurocognitive outcomes, and long-term functional impact remain under-represented. With the rapidly increasing amount of scientific information, systematization and literature review become particularly relevant. The findings may serve as a basis for shaping future research strategies aimed at deepening our understanding of the pathogenesis, diagnosis, and treatment of postural conditions in children.
